# Supplementary material for: Whose research benefits more from Twitter? On Twitter-worthiness of communication research and its role in reinforcing disparities of the field
Source: PLoS One. 2022 Dec 12;17(12):e0278840. doi: 10.1371/journal.pone.0278840 (PMC10045544; doi:10.1371/journal.pone.0278840)
Supplement: Attachment — Submitted filename: plos review.pdf [file pone.0278840.s001.pdf]

Whose research benefits more from Twitter? On Twitter-worthiness of communication research and its role in reinforcing disparities of the field.

Introduction -

“Social media might in turn become an enabler of the Matthew effect”

Explain specifically what is meant by “Matthew effect” and how social media can enable it.

“The central question of this study is: Whose research benefits Twitter more? And then we ask: Can these benefits translate into actual citations?”

Is this supposed to be “Whose research benefits **from** Twitter more”? In general the introduction is quite brief and could go into more detail about why this topic is important.

Data collection & Operationalizations of variables -

“The authors’ countries of origin were extracted”

How? Presumably not manually.

“Using a topic modeling technique”

Can you please give more information about this technique i.e. apart from using a dictionary, what is the algorithm?

“because there are many social factors associated with citation behaviors.”

For example?

Statistical Analysis -

This section is very succinct and could use much more elaboration. For example there appear to be 4 independent variables, which are a mixture of categorical (high impact, open access, geographic) and continuous (Topic membership θ), as predictors of citation/mention rate, but the number of variables is written as some unknown k and different symbols (G11, OA etc.) are used later in the paper where ‘x’ is used in this section.

"For our RQ1, we add interaction terms between factors determining twitterworthiness and region to all models"

Equations 1 and 3 are all rather trivial manipulations of each other and yet the model described in the quote above is not given.

The model used to produce table 3 does not appear to be described and should be.

Results -

"We observe a very long-tail distribution of Twitter mentions."

Please include this figure if you are going to mention it.

"As three is the upper limit of the IQR for Twitter mentions, we use it in the 282 following two figures to define papers with high Twitter mentions"

The upper limit is 4 in the G11 column for the mentions value.

Figure 2

Does the population of a country play a role in citation/mention behaviour? Many social media analyses find a bias towards mentioning other users who are geographically closer (e.g. Han, S.Y., Tsou, M.H. and Clarke, K.C., 2018. Revisiting the death of geography in the era of Big Data: The friction of distance in cyberspace and real space. *International Journal of Digital Earth*, *11*(5), pp.451-469).

The popularity of Twitter in a country also must play a huge factor in this analysis? E.g. in eastern Europe or China (i.e. much of the non-G12 category) Twitter is not very popular. Indeed, Twitter is officially blocked in China. Perhaps some other social media network is used there to disseminate research findings.

Table 3

Are all of the values statistically significant? e.g. OA*G11 , OA*US

Discussion -

Twitter has not been consistently popular since 2007 (https://trends.google.com/trends/explore?date=all&q=Twitter) and each country will have its own trajectory for Twitter use. The model does not account for this and it

would be useful to see either robustness checks (e.g. a subset analysis of a shorter period when Twitter use is approximately stable) or at least some discussion of this issue.

There is highly likely to be interplay between 1. Relative popularity of Twitter in US/G11 nations (measured by something like users per capita) 2. Number of communications studies researchers 3. Preference for mentioning/citing researchers geographically and culturally closer to the author. This warrants some discussion.

Conclusions -

"Additionally, this study investigated to what extent Twitter visiblity improves paper citations"

This needs clarification, where was the direction of causality established? A fit of y = mx+c (cf. equation 3) only establishes correlation between x and y.

General:

A scan of the whole MS for English/grammar is necessary e.g. "such as colonialism influence" -> "such as the influence of colonialism".

I don't doubt the findings of the paper and the methods are appropriate, however 1. A more thorough description of those methods is required 2. More robustness checks of the results are needed and 3. Recognition of the study's limitations, especially with respect to the unknown effect of inhomogeneous Twitter data.
