# Supplementary material for: Whose research benefits more from Twitter? On Twitter-worthiness of communication research and its role in reinforcing disparities of the field
Source: PLoS One. 2022 Dec 12;17(12):e0278840. doi: 10.1371/journal.pone.0278840 (PMC10045544; doi:10.1371/journal.pone.0278840)
Supplement: Attachment — Submitted filename: Twiterworthiness R2 Action.pdf [file pone.0278840.s003.pdf]

Dear editor and dear reviewers,

Thank you for your insightful feedback on our research paper “Whose research benefits more from Twitter? On Twitter-worthiness of communication research and its role in reinforcing disparities of the field.” (PONE-D-21-23079R1). Following the reviewers’ suggestions, we have revised the manuscript. We respond below through a point-by-point outline.

We hope that the revised manuscript will now be suited for publication in *PLOS ONE*. We are looking forward to hearing back from you.

Sincerely,
The Authors

Reviewer #3

Comment 1.1 *The authors have addressed the reviewers' comments fairly. The previous version's tone was too strong to be published. I believe the present version is adequate. However, several topics must still be addressed in the discussions. Communication journals, for example, with non-Western offices are uncommon. As a result, there is a discrepancy among the sampled articles.*
Response 1.1: We would like to thank the reviewer for acknowledging the improvement made in our previous revision, and for pointing out issues concerning the differences between journals (e.g. journals with or without non-Western offices). We fully agree with the point raised by the reviewer. We did model that in our Bayesian models with the varying intercept. In the revised manuscript we have strengthened this point in lines 302-5:
“There might be individual differences in μ and v according to characteristics of journals, e.g. countries of origin [65]. By adding a varying intercept according to the j-th journal as u_{oj} to model the individual differences in μ and v, our two models are expressed as….”

Comment 1.2 *Furthermore, papers dealing with specific concerns tend to receive more attention.*
Response 1.2: We agree with the reviewer that studies related to some concerns (e.g. Covid, as the papers suggested by the reviewer) may receive more social media engagements than others. Our paper has taken it into consideration and we modelled it as “Hot research topic” (θ_t). As shown in the revised manuscript (Lines 105-111), our conceptualization of twitter-worthiness has also incorporated this point. However, because our dataset was collected in 2019, the current analysis cannot capture new research concerns such as Covid. We have admitted this as a weakness in lines 470-3 and cited the important work by Chong & Park (2021):

“Third, the data used in this study is cross-sectional and count-based, with both the Twitter mentions and citations obtained in 2019. Although the cross-sectional dataset is easier to analyze, it presents three problems. The first problem is that our dataset doesn’t contain papers concerning current hot research topics which have been found to affect v, such as COVID-19 [73].”

Comment 1.3 *You might want to look into the following works.*

- *Park, H.C., Youn, J.H., & Park, H.W.(2018). Global mapping of scientific information exchange using altmetric data. Quality & Quantity. 53(2), 935–955.*
- *Holmberg,K., & Park,H.W.(2018). An altmetric investigation of the online visibility of South Korea-based scientific journals, Scientometrics. 117(1), 603–613*
- *Chong,M., & Park,H.W.(2021). COVID-19 in the Twitterverse, from epidemic to pandemic: information-sharing behavior and Twitter as an information carrier. Scientometrics.*
- *Park, H.J., Biddix, J.P., & Park, H.W. (2021). Discussion, news information, and research sharing on social media at the onset of Covid-19. EL PROFESIONAL DE LA INFORMACIÓN,v.30, n.4, e300405. 1-15.*

Response 1.3 We thank the reviewer for recommending the above references. We find them helpful and have incorporated them in the revised manuscript.

Reviewer #4

Comment 2.1 *The findings are very interesting and relevant to the area of information metric studies and research evaluation, but there is an important lack of information since the point of view of methods: You do not explain the software, method or code used to do the mining of Twitter, in order to recover all Twitter mentions. Even if you used WoS data to obtain this variable, you must explain it.*

Response 2.1 We would like to thank the reviewer for finding this study interesting and relevant. We agree with the reviewer that we could have provided more clarification on how Twitter-related data was collected. We have now added information about the software and method of our Twitter data in lines 207-211: ‘Altmetric, according to its website, “tracks Twitter attention in real-time via an API ... collect[s] tweets, retweets, and quoted tweets that contain a direct link to a scholarly output.” [57] Employing the Altmetric API and the R package rAltmetric [58], we extracted the mention counts of all 32187 articles in June 2019, with the DOIs.’
